# Mov10, an APOBEC3G-interacting RNA-binding protein, inhibits HIV-1 infection

**DOI:** 10.1186/1742-4690-8-S2-P2

**Published:** 2011-10-03

**Authors:** Shetal Arjan, Chad Swanson, Nathan Sherer, Michael Malim

**Affiliations:** 1 Department of Infectious Diseases, King's College London, London SE1 9RT, UK

## Background

Identification of cellular factors that negatively or positively regulate HIV-1 replication is essential to understand HIV-1 pathogenesis for development of novel antiviral treatments. The human cytidine deaminase APOBEC3G (A3G) is an intrinsic antiviral factor important for the host-mediated defence against HIV-1. We and others recently identified a panel of A3G-interacting RNA-binding proteins (RBPs) that focused our search for novel HIV-1 cofactors and restriction factors onto these proteins. Mov10, a superfamily-1 putative RNA helicase, interacts with A3G in an RNA-dependent manner. Mov10 associates with the RNA-induced silencing complex pathway and localises to mRNA processing bodies (PBs). Furthermore, orthologs of Mov10 in *Arabidopsis thaliana* and Drosophila melanogaster are necessary for antiviral small RNA-mediated silencing. Thus, we investigated whether Mov10 restricts HIV-1 replication.

## Materials and methods

The effect of Mov10 on HIV-1 virus production and ínfectivity was determined by ectopic expression and silencing of Mov10 in virus-producing HeLa and 293T cell lines. Cells were transfected with a plasmid encoding the full-length HIV-1_NL4-3_ provirus or infected with VSV-G pseudotyped HIV-1_NL4-3_ virus. Virion production was measured by p24^Gag^ ELISA and infectivity was determined by infecting a TZM reporter cell line with virus normalised by p24^Gag^ concentration.

## Results

The ectopic expression of Mov10 substantially decreased HIV-1 virion infectivity, whilst moderately decreasing virus production. Mov10 induced a significant reduction in the accumulation of minus strand strong stop DNA in the target cell, which likely accounted for the considerable loss in virion infectivity. Preliminary results showed a small decrease in HIV-1 genome incorporation into budding virions that may have contributed to the defect at strong stop. Mov10 comprises a 495 residue amino-terminal domain with no known protein motifs, and a 508 residue carboxy-terminal putative RNA helicase domain. To begin to identify important attributes in Mov10 we mutated known helicase motifs in the full-length protein; overexpression of these mutants determined that the carboxy-terminal putative RNA helicase domain of Mov10 is necessary for its antiviral activity. Furthermore, RNAi-mediated silencing of endogenous Mov10 moderately enhanced HIV-1 virion production with no discernable effect on virion infectivity. We also tested A3G antiviral activity in the context of Mov10 silencing, which was unaffected.

## Conclusions

Our findings indicate a potential antiviral role for Mov10 in the HIV-1 life cycle, independent of A3G antiviral activity. The effect of Mov10 silencing on HIV-1 was modest, however, this is currently being addressed in primary CD4^+^T cells and macrophages, the natural targets of HIV-1 infection. Furthermore, we are investigating whether Mov10 is upregulated by type I interferon and identifying molecular mechanism/s by which Mov10 is able to inhibit HIV-1 infection.

